# Comparison of Trans-septal Suturing Technique With Polyvinyl Alcohol Sponge-Based Nasal Packing for Hemostasis in Septoplasty

**DOI:** 10.7759/cureus.25161

**Published:** 2022-05-20

**Authors:** Rukamani Meena, Raman Sharma, Vikas Malhotra, Praveen K Rathore

**Affiliations:** 1 Otolaryngology - Head and Neck Surgery, Maulana Azad Medical College, New Delhi, IND

**Keywords:** nasal bleed, trans-septal suturing, anterior nasal packing, septoplasty, deviated nasal septum

## Abstract

Introduction

Despite the theoretical advantages of bleeding control, there is increased morbidity in postoperative pain, sleep disturbance, allergy, toxic shock syndrome, and mucosal injury with the nasal packing in septoplasty procedure for deviated nasal septum. Trans-septal suturing after septoplasty has been advocated as an effective alternative to conventional nasal packing. The current study aims to compare the frequency of subjective symptoms, such as postoperative nasal pain, nasal bleeding, postnasal drip, sleep disturbance, dysphagia, headache, and epiphora between the trans-septal suturing technique and nasal packing in septoplasty surgery.

Methods

We prospectively recruited all adult septoplasty patients for a one-year duration. Trans-septal nasal suturing was done for hemostasis after septoplasty in the case group. Anterior nasal packing after septoplasty was performed in the control group. The postoperative subjective symptoms were evaluated, such as postoperative nasal pain, nasal bleeding, postnasal drip, sleep disturbance, dysphagia, headache, and epiphora. Procedure-related complications were compared between the two groups.

Results

A total of 50 patients were recruited for the study (25 in each group). The postoperative symptoms evaluation suggested that the number of patients with postoperative pain was significantly higher in the control group on both occasions. Besides pain, a significantly higher number of patients in the control group had symptoms of nasal bleeding, postnasal drip, sleep disturbance, dysphagia, headache, and epiphora.

Conclusion

Trans-septal suture technique is an effective alternative to nasal packing with a low risk of nasal pain, bleeding, postnasal drip, epiphora, headache, dysphagia, and sleep disturbance. In addition, there is a low risk of complications like nasal bleeding, septal hematoma, septal perforation, and synechiae formation. The only disadvantage of trans-septal suturing compared to PVA-coated nasal packing is the increase in the operative time.

## Introduction

Deviated nasal septum (DNS) has a prevalence of 75%-80% in the general population [1]. The deformity can be classified into spurs, deviations, and dislocations [2]. The clinical symptoms include nasal obstruction, headache, sinusitis, epistaxis, anosmia, and sometimes an external deformity. Septoplasty has been recommended to correct such deformities either alone or in combination with inferior turbinoplasty, endoscopic sinus surgery (ESS), and rhinoplasty [3]. The postoperative packing of the nasal cavity minimizes the chances of postoperative bleeding, mucosal adhesions, recurrence, or persistence of septal deviation [4]. Various materials have been used for packings, such as paraffin gauze, Vaseline gauze, glove fingers, silastic sheets, gel foam, polyvinyl alcohol (PVA) coated packings, antibiotics impregnated gauge, and fibrin glue [5]. Despite the theoretical advantages of bleeding control, there is increased morbidity in postoperative pain, sleep disturbance, allergy, toxic shock syndrome (TSS), and mucosal injury [5-7]. Lee et al. [8] devised a concept of continuous hemostatic-stabilizing trans-septal suturing after septoplasty. They concluded it to be a useful modification with only a minor increase in operating time. The current evidence concerning the effectiveness of trans-septal suturing over nasal packing in postoperative patient comfort after septoplasty is limited [9,10]. The current study aims to compare the frequency of subjective symptoms, such as postoperative nasal pain, nasal bleeding, postnasal drip, sleep disturbance, dysphagia, headache, and epiphora between the trans-septal suturing technique and nasal packing in septoplasty surgery.

## Materials and methods

This study was prospectively conducted at a tertiary care center associated with a teaching medical college in New Delhi, India, for a one-year duration after approval from the institutional ethical committee (Institutional Ethics Committee of Maulana Azad Medical College, New Delhi; approval number: Thesis S.No.10/IEC/MAMC/2016). We recruited all adult patients with nasal obstruction symptoms and planned for septoplasty during the study period. Following the septoplasty procedure, trans-septal nasal suturing was done for hemostasis (case group). Additional age and gender-matched subjects with similar symptoms but treated with anterior PVA nasal packing after septoplasty (control group) were enrolled in the study for comparison. Patients with medical comorbidities and bleeding disorders were excluded from the study. One control subject was there for each patient in the case group. The subjective symptoms were evaluated, such as postoperative nasal pain using a visual analog scale (VAS) ( 1-3 minimal; 4-6 moderate; 7-10 severe pain), nasal bleeding, postnasal drip, sleep disturbance, dysphagia, headache, and epiphora. Patients were interviewed regarding their symptoms on the first and second postoperative days. One week postoperatively, patients were assessed for septal hematoma, nasal discharge, and nasal crusting.

Further, at four weeks period, patients were evaluated for synechiae, septal perforation, and nasal crusting. The surgical durations for septoplasty surgeries in both groups were compared. The categorical variables were expressed as frequency and percentages, and the continuous variables were expressed as mean±standard deviation. The categorical variables were compared using the Fisher exact test, and the continuous variables were compared using the independent samples t-test. We used IBM Statistical Package for the Social Sciences software for Windows, Version 22.0 (IBM Corp., Armonk, NY) for statistical analysis. A p-value of <0.05 was considered statistically significant.

## Results

A total of 50 patients were recruited for the study (25 in each group). There were 19 males and six females, each in the case and matched control groups. The mean age was 26.6±8.2 years. The average operative time in the case group (40.72±4.9 minutes) was significantly longer than in the control group (48.40±5.9 minutes). The postoperative symptoms evaluation suggested that the number of patients with postoperative pain was significantly higher in the control group on the first and second postoperative days (p < 0.05). The detailed distribution is provided in Table 1.

**Table 1:** Postoperative pain among the case (trans-septal suturing) and control (nasal packing) groups.

Pain visual analog scale	First postoperative day		Second Postoperative day	
	Case group, n (%)	Control group, n (%)	Case group, n (%)	Control group, n (%)
Mild (1-3)	2 (8%)	0 (0%)	2 (8%)	10 (40%)
Moderate (4-6)	0 (0%)	18 (72%)	0 (0%)	13 (52%)
Severe (7-10)	0 (0%)	7 (28%)	0 (0%)	2 (8%)

Besides pain, a significantly higher number of patients in the control group had symptoms of nasal bleeding, postnasal drip, sleep disturbance, dysphagia, headache, and epiphora. The details are presented in Table 2. Most symptoms were observed on the first postoperative day.

**Table 2:** Postoperative clinical symptoms among the case (trans-septal suturing) and control (nasal packing) groups.

Clinical symptoms	Postoperative day	Case group, n (%)	Control group, n (%)	P-value
Nasal bleeding	First postoperative day	2 (8%)	0 (0%)	0.49
	Second postoperative day	0 (0%)	6 (24%)	< 0.05
Postnasal drip	First postoperative day	0 (0%)	23 (92%)	< 0.05
	Second postoperative day	0 (0%)	0 (0%)	1
Sleep disturbance	First postoperative day	4 (16%)	20 (80%)	< 0.05
	Second postoperative day	0 (0%)	0 (0%)	1
Dysphagia	First postoperative day	1 (4%)	24 (96%)	< 0.05
	Second postoperative day	0 (0%)	0 (0%)	1
Headache	First postoperative day	3 (12%)	23 (92%)	< 0.05
	Second postoperative day	0 (0%)	25 (100%)	< 0.05
Epiphora	First postoperative day	0 (0%)	23 (92%)	< 0.05
	Second postoperative day	0 (0%)	0 (0%)	1

There were no statistically significant differences between the two groups in first week and fourth week regarding the late symptoms and complications related to the septoplasty procedure. The detailed results are provided in Table 3.

**Table 3:** Procedure-related complications among the case (trans-septal suturing) and control (nasal packing) groups.

Duration	Symptoms	Case group, n (%)	Control group, n (%)	P-value
First week	Hematoma	0 (0%)	0 (0%)	1
	Nasal discharge	18 (72%)	16 (64%)	0.76
	Nasal crusting	13 (52%)	15 (60%)	0.77
Four weeks	Synechiae	0 (0%)	2 (8%)	0.48
	Septal perforation	0 (0%)	0 (0%)	1
	Nasal crusting	8 (32%)	12 (48%)	0.38

## Discussion

Nasal packing has been historically used in septoplasty to prevent bleeding, and hematoma perforation and to stabilize the cartilage and bony skeleton. Trans-septal suture technique has been advocated as a valid and comfortable alternative for packing. Our study supports the use of the trans-septal suture technique as a better method for patients’ postoperative comfort. The patients in our study belonged to a younger age group with a mean age of younger than 30 years. This could probably be related to a higher risk of accidents and traumas that result in an increased incidence of septal deviation in young individuals. The level of postoperative nasal pain on both the first and second postoperative days was higher among the packing group than in the trans-septal suturing group.

In our study, all patients in the nasal packing group had severe pain on the first and second postoperative days, while only a few suturing group patients had mild pain. This finding is in line with the study conducted by Naghibzadeh et al. [7], Waliker et al. [11], and Awan et al. [12]. Postoperative bleeding in the packing group occurred in five patients on the second postoperative day after nasal pack removal. While in the trans-septal suturing group, two patients had minimal bleeding on the first postoperative day and no bleeding on the second postoperative day. Naghibzadeh et al. [7] reported only three cases that developed postoperative hemorrhage, two from the non-packing group and one from the packing group. Postnasal drip was also frequent with the packing, probably due to aggravated response of the nasal mucosa to the nasal pack. These results were consistent with the study by Said et al. [9]. There were significant sleep disturbances and dysphagia on the first postoperative day in the packing group compared to the suturing group, consistent with the results of Turhan et al. [13] and Korkut et al. [14], respectively. If a patient swallows with the nasal passages blocked (Toynbee maneuver), air cannot pass anteriorly and is insufflated into the middle ear. This unpleasant feeling results in poor oral intake while the nasal packing is in place. Our results are similar to the studies by Waliker et al. [11] and Jawaid et al. [15] regarding postoperative headache and epiphora, which were more profound on the first postoperative day in the packing group. Epiphora is mainly due to obstruction to the nasolacrimal duct outflow. The septal hematoma was not there in any case of our study. This could be because of using a PVA nasal pack which prevented the accumulation of blood in the septum. In contrast, in the suturing group, the trans-septal suture prevented the accumulation of blood in the septum. In similar studies, very few cases that developed septal hematoma have been reported [9,16]. It has been found that packing makes the nasal mucosa raw and more susceptible to synechiae formation. Adhesions can be prevented by avoiding nasal packing, careful handling of the septal mucosa, avoiding manipulation of the turbinates, and the meticulous placement of instruments in the surgical site [17]. In our findings, two patients developed synechiae in the packing group, consistent with the other studies [7]. No septal perforation was documented in the current study; the results were similar to other studies [7], except for the findings of Cukurova et al. [18], where eight cases in suturing group and 11 cases in the packing group developed perforation out of 697 patients. The main cause of septal perforation includes careless surgery, pressure necrosis by the bulky nasal pack, or using a needle and suture of unusual thickness. Nasal crusting was present in a comparable number of patients in each group, which could be due to ineffective or insufficient nasal lavage. The average operative time in the suturing group was higher than the packing group, and the difference was statically significant. The results were similar to Özkırış et al. [19]. They concluded that using the trans-septal suturing technique is a useful alternative to packing, with only a minor increase in operating time. Wang et al. [20] performed a systematic review of nasal packing effect-related outcomes compared to nasal septum sutures among septoplasty patients. They concluded that there is a possibility of adopting nasal septal suturing as an alternative to nasal packing after septoplasty. However, the need for well-designed studies was also suggested.

The current study has some limitations. First, the study has a limited sample size of 50 patients. This could be because of the one-year recruitment period of the study. Second, the study does not analyze the patients and surgeon-related factors that could potentially influence the outcomes of two procedures. Third, the current study analyzed only the early postoperative outcomes, i.e., up to four weeks. The long-term outcomes and complications were not discussed. Still, the age and gender-matched comparison groups without medical comorbidities standardize the baseline parameters to some extent and can help predict the procedure's effectiveness better.

## Conclusions

Trans-septal suture technique is an effective alternative to nasal packing with minimal risk of nasal pain, bleeding, postnasal drip, epiphora, headache, dysphagia, and sleep disturbance. In addition, there is a low risk of complications like nasal bleeding, septal hematoma, septal perforation, and synechiae formation. On contrary, nasal packing is associated with significantly higher patient discomfort in the early postoperative period. The only disadvantage of trans-septal suturing compared to PVA-coated nasal packing is the increase in the operative time. Further, well-planned prospective studies will help in strengthening this evidence.
